# Temporal Orienting and Alerting – The Same or Different?

**DOI:** 10.3389/fpsyg.2012.00236

**Published:** 2012-07-11

**Authors:** Noam Weinbach, Avishai Henik

**Affiliations:** ^1^Department of Psychology and Zlotowski Center for Neuroscience, Ben-Gurion University of the NegevBeer-Sheva, Israel

Attention helps regulate what to attend to and what to filter out. A warning cue prior to an event can be used to direct attention and improve performance when response to an imperative target is required. Various studies have suggested that warning cues may induce a change in alertness or modulate temporal anticipation of an upcoming event. The current literature presents similar effects for these two functions; hence, effects of a warning cue are sometimes attributed to changes in the state of alertness and in other cases, to voluntary orienting of attention in time. In this article we will discuss whether temporal orienting of attention and alertness are dissociable.

## Temporal Orienting

Spatial orienting of attention (Posner et al., 1980) has been studied for many years. Recently, studies demonstrated the ability to flexibly and voluntarily orient attention to moments in time – temporal orienting of attention (Coull and Nobre, 1998; for review see Correa, 2010). Many studies on voluntary temporal orienting present symbolic warning cues prior to a target that predict, with high probability, the specific time of target onset. For example, a red rectangle can be used to predict with 75% chance that the target will appear shortly, following 400 ms, and a green rectangle can be used to predict with 75% chance that the target will appear later – following 1,300 ms. The cues are considered valid when the target appears at the predicted time (i.e., 75% of the trials), and invalid when the target appears at a temporally unexpected time (i.e., 25% of the trials). Reaction times (RTs) are faster following valid cues compared with invalid cues. Another method used to study temporal preparation is manipulating the time interval between the warning cue and the target (i.e., foreperiod). For example, when using a constant foreperiod in a block of trials (i.e., within a block the target always appears following the same foreperiod), RTs will be faster for a shorter foreperiod block (e.g., 800 ms) compared with a long foreperiod block (e.g., 2,000 ms, see Rolke and Hofmann, 2007, for a typical study). The time between the cue and target allows top-down temporal preparation to develop, but it will be less accurate as time is prolonged. In contrast, when different foreperiods are intermixed within a block, expectancy builds up as time elapses and performance will be better at later SOA's, a phenomenon called “*the foreperiod effect*” (see Niemi and Näätänen, 1981).

## Alertness

Alertness is considered by some researchers as an attentional system that helps regulate the intensity of attention to given stimuli (Posner and Petersen, 1990; Sturm et al., 1999). Effects of alertness are attributed to a high state of arousal for a short period of time following an abrupt external event (i.e., phasic alertness). Most studies use neutral warning cues (i.e., task-irrelevant) prior to a target to induce a state of alertness. Faster RTs are observed following these cues compared with a no-cue condition, in which arousal is low. It was argued that a warning cue that elevates alertness has the optimal influence on performance at a foreperiod of 500 ms (Posner and Boies, 1971). Most studies on alerting cues use foreperiods that range roughly between 100–800 ms. Some authors use the term “accessory stimuli” rather than warning cues if the foreperiod is less than 500 ms (Hackley et al., 2009).

## The Problem

Although neutral warning signals do not necessarily predict the exact onset time of the target, they may still trigger temporal expectation by indicating that a target will appear shortly. On the other hand, temporal orienting cues, which trigger voluntary modulation of attention in time, also involve, to some extent, a change in the state of alertness (Correa et al., 2004). Since both processes can be triggered by a single cue, there is difficulty in assessing to which extent the effects following a warning cue reflect benefit due to bottom-up arousal or are due to top-down temporal expectancy. It makes sense that the shorter the interval between the warning cue and the target, the less likely it is for top-down processes to develop. However, how short is “short”? Studies on phasic alerting show that alerting cues can reduce RTs even at a foreperiod of 100 ms (e.g., Fernandez-Duque and Posner, 1997). Some argue that at foreperiods below 500 ms, temporal expectancy cannot build up (Hackley et al., 2009). However, other studies on temporal preparation challenge this view and report temporal preparation effects even at foreperiods of 200 ms when manipulating temporal contingencies (Thomaschke et al., 2011) or 300 ms for effects of symbolic temporal cues (Coull and Nobre, 1998). Clearly, there is an overlap in the time course of temporal preparation and alerting.

In addition to the methodological difficulty in dissociating these effects, the literature can be sometimes confusing when considering the definitions different researchers use for alerting and temporal orienting. In fact, some authors define alerting basically in the same way as temporal orienting is defined. For example, “…*alerting is the ability to make use of a cue which provides information about the onset time of a target stimulus, and thus triggers the allocation of attention at a given point in time*” (Dye et al., 2009, p. 1780). Others use a more general definition for alerting, which does not necessarily consider temporal expectancy. For example, “… *the ability to increase response readiness for a short period of time subsequent to external cues or stimuli (phasic alertness)*” (Sturm and Willmes, 2001, p. S76).

Because of the overlap in definitions and due to the overlap in time-course of the effects as mentioned above, it is not surprising that many studies report significantly similar findings regarding the behavioral and neuronal features of the two processes. These findings are attributed to alerting in some studies and in others, to temporal orienting.

## Comparing Some of the Effects of Alerting and Temporal Orienting

Both alerting and temporal orienting cues usually produce faster motor execution of response to an imperative target compared with no-cue or invalid temporal cue conditions, respectively. A major question was whether the source of faster execution of response could be attributed only to motor preparation, or was there also a change in early perceptual and response selection processing stages? Event-related potential (ERP) studies have demonstrated that both alerting and voluntary temporal orienting modulate similar components that are related to early processing stages such as perceptual and response selection, rather than just late motor preparation (for alerting see Hackley and Valle-Inclán, 1998; Böckler et al., 2011; for temporal orienting see Correa et al., 2006a; Lange et al., 2006). This is also supported by behavioral studies demonstrating that temporal preparation can improve perceptual processing by operating at the onset of sensory information accumulation, facilitating perceptual discrimination, improving perceptual sensitivity and discrimination accuracy (e.g., Correa et al., 2005, 2006b; Rolke and Hofmann, 2007; Rolke, 2008; Seibold et al., 2011). Phasic alerting has also been found to increase perceptual processing speed, improve conscious perception, and bias perceptual processing (Matthias et al., 2010; Kusnir et al., 2011; Weinbach and Henik, 2011; Finke et al., 2012).With regard to response selection processes, both alerting and temporal orienting have been found to have similar effects. For example, alerting cues have been suggested to increase response conflict due to increased activation of the stimulus-response link (Fischer et al., 2010, 2012). Similarly, valid temporal cues have been suggested to disrupt response selection by automatically activating competing responses (Correa et al., 2010).

From a neurophysiological aspect, both the effects of alerting and of temporal cueing can be reduced by drugs such as Clonidine, which reduce norepinephrine (NE) release (Coull et al., 2001). However, the brain activity that accompanied the use of Clonidine in alerting vs. temporal orienting cues did not overlap, and it was argued that modulation of the alerting effect by Clonidine is unlikely to be due to an underlying effect on temporal orienting processes (Coull et al., 2001).

In addition, when reviewing the literature on the neural correlates of temporal orienting cues and alerting cues, a clear dissociation between them is also somewhat difficult to find. Studies show that both alerting cues and temporal orienting cues are associated with similar regional activity in the left hemisphere (Coull et al., 2001; Fan et al., 2005). This led Coull et al. to conclude that “… *alerting effect primarily indexes temporal orienting and motor preparation, rather than arousal or phasic alertness*” (p. 81). However, it is important to note that there is an ongoing debate regarding the lateralization of alerting and some uncertainties remain (Petersen and Posner, 2012).

In summary, all of the above are examples that could indicate that alerting and temporal preparation actually represent the same preparation process.

## Dissociating Alerting and Temporal Orienting

The difficulty in dissociating the effects of alerting and temporal orienting could be because they both actually reflect the same process of preparation. Alternatively, they could represent different processes that function similarly. There are two main problems in dissociating alerting and temporal orienting; one is methodological and the second is their definitions. Methodologically, both processes can be triggered by the same warning cue and overlap in time-course. Regarding their definitions, different authors sometimes define both processes in a similar way and this could lead to similar operationalization. However, it is important to note that there is evidence that these processes are dissociable.

At first, a reduction in RTs following warning cues is observed even when the cues are not temporally predictive. When making sure that following the warning cue, there is equal probability for the target to appear at each foreperiod (a technique called “*non-aging foreperiods*,” see Niemi and Näätänen, 1981) there is still reduction in RTs following the warning cue (Whitehead, 1991). This benefit cannot be understood by reduction of uncertainty regarding the temporal onset of the target (Fernandez-Duque and Posner, 1997). In addition, arousing cues (i.e., accessory stimuli) that are presented concurrently or even following the target still produce a benefit in RTs (e.g., Stahl and Rammsayer, 2005; Kiesel and Miller, 2007), even though there is no temporal preparation in this situation. In a more recent study, Hackley et al. (2009) reported a dissociation between phasic alerting and temporal expectancy after showing that alerting cues still induce benefit in RTs even when participants know in advance exactly when the target will appear, making the alerting cues completely task-irrelevant. Even more recently, Lawrence and Klein (in press) offered a clever methodological solution for examining the pure effect of alerting by demonstrating the benefit of these cues in a block of trials where there were absolutely no contingencies between the alerting signals and the target.

These examples of dissociations between the two processes lead to the conclusion that alerting and temporal orienting represent different processes.

## Conclusion and Practical Suggestions

We suggest a distinction between temporal orienting, in which temporal information is inherent in the cues, and arousal, which does not depend on temporal contingencies. Both types of preparation can be achieved voluntarily or automatically. Voluntary temporal orienting is best reflected by tasks using symbolic temporal cues (Coull and Nobre, 1998). Automatic temporal orienting can be observed following regular rhythms that orient attention in time involuntarily (Rohenkohl et al., 2011). Automatic arousal is reflected in phasic alertness, which has the largest effect at short foreperiods and can occur independently of temporal contingencies. Voluntary arousal is what authors name “tonic alertness,” meaning the general ability to stay alert and prepared for detecting infrequent stimuli during a task (usually measured in vigilance and continuous performance tasks).

The distinction between arousal and temporal orienting should be taken into consideration when studying temporal preparation and alerting (arousal) because these processes are commonly confounded in most experimental designs. In order to examine one process only, it is necessary to control for the irrelevant process. Researchers can adopt techniques such as non-aging foreperiod distribution in order to control strategic temporal expectancy processes following the alerting cue. Note that Lawrence and Klein (in press) have recently suggested another methodology in order to reveal a pure effect of alerting cues.

Studies on cued temporal orienting can include non-informative neutral cues, which can be considered as the baseline arousal level, and compare the results achieved with these cues to those with valid cueing (i.e., high temporal expectation) and invalid cueing (i.e., low temporal expectation; see similar procedure in Coull and Nobre, 1998; Coull et al., 2001).
